# Analysis of Re-visits to the Psychiatric Emergency Department in a Tertiary Public Hospital in Spain According to Socioeconomic Status

**DOI:** 10.1192/j.eurpsy.2026.11334

**Published:** 2026-07-31

**Authors:** A. G. P. Garcia De Olalla, M. A. Irigaray, N. L. San Pelayo, I. B. Goikoetxea, A. A. Trueba, M. A. G. Torres

**Affiliations:** Psychiatry, Osakidetza, Bilbao, Spain

## Abstract

**Introduction:**

Socioeconomic status plays a critical role in shaping mental health outcomes and access to care. Individuals with lower socioeconomis status may experience greater psychosocial stress and reduced availability of community resources, potentially increasing reliance on emergency psychiatric services. Re-visits to psychiatric emergency departments are an important indicator of care continuity and unmet needs. However, evidence linking socioeconomic status to psychiatric re-attendance within public healthcare systems remains limited. This study examines whether socioeconomic status is associated with re-visits to a psychiatric emergency department in a tertiary public hospital in Spain, aiming to identify potential disparities in service use.

**Objectives:**

To examine whether individual socioeconomic status influences the number of re-visits to a public psychiatric emergency department.

**Methods:**

Data from 1,800 emergency psychiatric visits between January and May 2024 from Basurto University Hospital (Bilbao) were collected. Each individual was assigned an approximate socioeconomic status based on the average disposable household income of their residential neighborhood within the hospital’s catchment health region (999 cases, corresponding to 1,412 visits). A logistic regression model was then applied, considering visits by disposable household income as the dependent variable. The re-visit rate was calculated by dividing the number of re-visits by the total number of visits during the five-month study period, at 7 days, and at 72 hours following the initial presentation.

**Results:**

A total of 27.1% of patients attended the psychiatric emergency department more than once during the study period; among them, 22% resided within the study’s health region (the remainder corresponded to individuals without official census registration or residing in other health regions). Within this 22%, 7.2% re-attended the emergency department within 72 hours. Re-visits were more frequent among individuals with low income (24.7%) compared to those with middle (22.0%) and high income (14.1%) (p=0.01), both at 7 days and at 3 days. The likelihood of re-visit was 40% higher among patients from low-income neighborhoods (adjusted OR = 1.40; 95% CI: 1.02–1.92).

**Conclusions:**

Lower socioeconomic status was associated with a significantly higher likelihood of re-visits to the psychiatric emergency department. Patients from low-income neighborhoods returned more frequently within both 72 hours and 7 days following initial assessment. These findings suggest potential disparities in continuity of care and access to adequate community mental health resources. Targeted interventions to strengthen outpatient support and follow-up for socioeconomically vulnerable populations may be needed to reduce emergency re-attendance and improve clinical outcomes.

**Disclosure of Interest:**

None Declared

